# Mild COVID-19 has no detrimental effect on semen quality

**DOI:** 10.1186/s12610-023-00190-2

**Published:** 2023-06-15

**Authors:** Philippos Edimiris, Cornelius Doehmen, Lisa Müller, Marcel Andrée, Dunja Maria Baston-Buest, Sebastian Buest, Ortwin Adams, Jan-Steffen Krüssel, Alexandra Petra Bielfeld

**Affiliations:** 1 Department of OB/GYN and REI (UniKiD), Medical Center, University of Duesseldorf, Moorenstr. 5, 40225 Duesseldorf, Germany; 2 Kinderwunschzentrum Niederrhein, Madrider Str. 6, 41069 Moenchengladbach, Germany; 3 Institute of Virology, University Hospital Duesseldorf, Heinrich Heine University Duesseldorf, Duesseldorf, Germany

**Keywords:** COVID-19, Sperm, SARS-CoV-2, Ejaculate, Pandemic, COVID-19, Sperme, SARS-CoV-2, Éjaculat, Pandémie

## Abstract

**Background:**

As of today, the effect of coronavirus disease 2019 (COVID-19) on male fertility remains unclear. Studies published so far have partly contradictory results, likely due to very small sample sizes and heterogeneous populations.

To gain a deeper understanding of the impact of COVID-19 on male fertility, we performed a prospective case–control study, in which we examined the ejaculate of 37 subjects, including 25 subjects in the acute phase of mild COVID-19 and 12 subjects who did not suffer from COVID-19. Determination of semen parameters, severe acute respiratory syndrome coronavirus type 2 (SARS-CoV-2) qPCR, and infectivity analysis were performed in the acute phase of the disease and in series.

**Results:**

Semen parameter values did not differ significantly between subjects with mild COVID-19 and the control group. The serial examination of semen parameters revealed no significant changes between 4, 18, and 82 days after the onset of symptoms. SARS-CoV-2 RNA or infectious particles could not be detected in any ejaculate.

**Conclusion:**

Mild COVID-19 seems to have no detrimental effect on semen parameter values.

## Résumé

**Contexte:**

À ce jour, l’effet de la maladie due au coronavirus 2019 (COVID-19) sur la fertilité masculine reste incertain. Les études publiées jusqu’à présent ont des résultats partiellement contradictoires, ce qui est probablement dû à la très petite taille des échantillons et l’hétérogénéité des populations. Pour mieux comprendre l’impact de la COVID-19 sur la fertilité masculine, nous avons réalisé une étude cas-témoins prospective, dans laquelle nous avons examiné l’éjaculat de 37 sujets, dont 25 sujets en phase aiguë de COVID-19 légère et 12 sujets qui ne souffraient pas de la COVID-19. La détermination des paramètres séminaux, la qPCR du coronavirus du syndrome respiratoire aigu sévère de type 2 (SRAS-CoV-2), et l’analyse de l’infectiosité ont été effectuées dans la phase aiguë de la maladie et dans la série.

**Résultats:**

Les valeurs des paramètres du sperme ne différaient pas significativement entre les hommes atteints de la COVID-19 légère et ceux du groupe témoin. L’examen en série des paramètres du sperme n’a révélé aucun changement significatif entre 4, 18 et 82 jours après l’apparition des symptômes. L’ARN du SARS-CoV-2 ou les particules infectieuses n’ont été détectés dans aucun des éjaculats.

**Conclusion:**

La COVID-19 de forme légère ne semble pas avoir d’effet néfaste sur les valeurs des paramètres du sperme.

## Background

Since the onset of the coronavirus disease 2019 (COVID-19) pandemic in December 2019, which is caused by the severe acute respiratory syndrome coronavirus (SARS-CoV-2), the World Health Organization (WHO) has reported nearly 640 million COVID-19 cases and 6.6 million deaths worldwide as of December 2022 [1, 2]. Infection with SARS-CoV-2 results from the interaction of the viral spike (S) protein and the angiotensin-converting enzyme 2 (ACE2) receptor to initiate glycoprotein-mediated entry into the cell [3]. Furthermore, the cellular serine protease, transmembrane protease serine 2 (TMPRSS2), facilitates proteolytic cleavage of the spike protein between the S1 and S2 subunits to initiate membrane fusion. ACE2 shows medium expression levels in the lungs, while it is highly expressed in renal tissue, cardiomyocytes, and testicular tissue [4, 5]. The complex expression pattern of ACE2 might contribute to the wide range of symptoms in patients infected with SARS-CoV-2 with mild to severe disease manifestation [6]. Pneumonia and acute respiratory distress syndrome are the major complications [7]. Since ACE2 receptors are also present in the testis, the potential impact of COVID-19 on the male reproductive system has been investigated from the beginning of the pandemic. Ejaculates have been tested for the presence of SARS-CoV-2 RNA and whether sperm parameters were affected by COVID-19 has also been investigated [8–21]; however, the results of the studies are partly contradictory, which may be explained by the different study time points and the different composition of the study population.

Consistent with our own data published in August 2020 [8], most studies excluded the presence of SARS-CoV-2 RNA in semen from both acutely infected [9–13, 22, 23] and recovered [8, 14, 15, 21, 23, 24] individuals.

However, three studies found SARS-CoV-2 RNA in 1/15 [16] and 4/15 [25] of acutely infected subjects, as well as 2/23 [25] and 1/43 [17] of recovered subjects. A meta-analysis showed that the stage of the disease is the only positive predictive factor for the detection of SARS-CoV-2 in the ejaculate [26]. The authors added that most of the available studies provide limited information on the method of semen collection and processing, and therefore, the possibility that the localization of SARS-CoV-2 in semen is due to possible contamination cannot be excluded [26].

In most studies, the authors assumed that COVID-19 had a detrimental effect on male fertility [8, 12, 14, 17]. For example, in the publication by Gacci et al., 25% of subjects presented oligo-, crypto-, or azoo-spermia [17]. While our previous study, and most other studies, found that the effect is related to disease severity [8, 17] another study demonstrated that the effect was independent of the course of disease [14]. There are different study results on the question of how long the impairment of sperm quality lasts. While one study showed that semen parameter changes are transient and return to normal three months after recovery [18], another demonstrated that sperm parameter deterioration still persisted three months after recovery [14].

Regarding the influence of vaccination against COVID-19, all researchers agree that it has no effect on sperm quality [27, 28]; however, whether vaccination prevents the above-described deterioration of sperm parameter values has not yet been investigated.

This study was designed to clarify whether even mild COVID-19 has an impact on sperm parameter values, how long this impact lasts, and whether vaccination can prevent deterioration of sperm quality. Therefore, we examined the ejaculate of subjects acutely infected with mild COVID-19, who were either vaccinated or unvaccinated, at three different time points: at symptom onset, two weeks later and 80 days later. At each time point, semen parameters and SARS-CoV-2 RNA were analyzed. To screen for infectious particles, semen samples obtained in the acute phase of infection were inoculated into Vero cell cultures.

## Material and methods

**Fig. 1:**
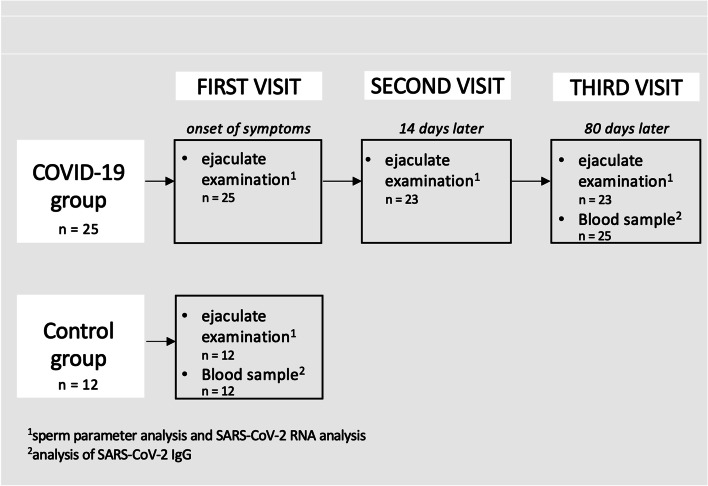
Study flow chart. Legend: Illustration of the study schedule for the COVID-19 subjects and for the subjects from the control group

The present work is a case–control study in accordance with the Declaration of Helsinki conducted at the Heinrich-Heine-University Hospital Interdisciplinary Fertility Center Duesseldorf UniKiD, in cooperation with the Institute of Virology of the Heinrich-Heine-University Duesseldorf, Germany. The subject recruitment was carried out from December 2020 to May 2022. An approval of the local ethics committee (2020–938) was issued. Written informed consent was obtained from each participating subject. Ejaculates from 25 subjects with acute COVID-19 infection and from a control population of 12 subjects were examined. In 23 subjects with acute COVID-19 infection, a series of three ejaculates were examined (Fig. 1).

The subjects were included after detection of SARS-CoV-2 from throat swab samples by reverse transcription-polymerase chain reaction (RT-PCR). Inclusion criteria for the case group with acute COVID-19 were: age ≥ 18 years, willingness to participate to the study, confirmed COVID-19 per RT-PCR, and a mild course of disease. The exclusion criterion was hospitalization due to COVID-19. In each of the two groups, there was one subject with a known male fertility disorder who had undergone infertility treatment as a result. In addition, there was one subject in each of the two groups who had a history of testicular carcinoma. The subject with testicular carcinoma in the control group had asthenozoospermia. The subject with testicular carcinoma in the COVID-19 group had normozoospermia, oligo-asthenozoospermia and teratozoospermia at the first, second and third visits, respectively. The study included three visits in which semen was collected. A blood sample was also taken at the third visit. Each ejaculate sample was tested for SARS-CoV-2 RNA. Sperm parameters were analyzed in each ejaculate. Blood was collected for the detection of antibodies against SARS-CoV-2. All men answered a routine questionnaire to obtain their clinical history. The ejaculate was collected by masturbation into a sterile cup. The first ejaculate, however, was obtained at home, since all subjects were still in quarantine at the first visit. The cup was transported to the investigation site in a styrofoam box within 60 min after semen collection. The second and third samples were obtained at the investigation site. At the first visit, participants were not asked to maintain a certain abstinence because it was more important to collect a sample in the acute phase of the infection. At the second and third visits, participants were asked to maintain an abstinence of 2–7 days.

Inclusion criteria for the control group were: age ≥ 18 years, willingness to participate to the study, and no COVID-19, which was verified by both a medical history and the determination of SARS-CoV-2 IgG in serum.

For the control group, only one visit was required, which included an ejaculate sample for analysis of SARS-CoV-2 and sperm parameters and a blood sample for the detection of antibodies against SARS-CoV-2. The ejaculate was collected at the study site.

Processing and examination of ejaculates was performed as described previously [27], using the 2010 WHO guideline laboratory manual for the examination and processing of human semen [29] by two highly experienced embryologists to control for consistency. The report included: ejaculate volume (ml), sperm concentration (number/ml), total sperm number, and sperm motility (%). Sperm motility was reported as progressive motility (%) and total motility (progressive and non-progressive motile sperm; %).

### SARS-CoV-2 analysis in semen

RNA was extracted from semen samples using the EZ1 Virus Mini Kit (Qiagen, Hilden, Germany) according to the manufacturers’ protocol. SARS-CoV-2 qRT-PCR analysis was by the AgPath-ID™ One-Step RT-PCR Kit (ThermoFisher Scientific, Waltham, MA, USA) on an Applied Biosystems™ 7500 FAST sequence detector system (PE Applied Biosystems, Weiterstadt, Germany) with the TaqMan™ qPCR Mastermixes LightMix Modular SARS and Wuhan CoV E-gene (TIB MolBiol, Berlin, Germany) and the LightMix® Modular EAV RNA Extraction Control (Roche, Basel, Switzerland). A 113 base-pair amplicon in the E-gene of SARS-CoV-2 was amplified and detected as described by Corman et al. [30]. The thermal protocol was shortened to 40 cycles of 95 °C [31].

Vero cells CCL-81™ (ATTC, Manassas, Virginia, USA) were seeded at 1.25 × 10^5^ cells per T25 flask in Dulbecco's Modified Eagle Medium (DMEM; ThermoFisher Scientific), 2% (v/v) fetal calf serum (PAN Biotech, Aidenbach, Germany), 100 U/ml penicillin, and 100 μg/ml streptomycin (Gibco™, ThermoFisher). Samples of semen (200 µl) were added to the cell culture after brief centrifugation (500 g, 5 min, RT). Cells were inspected for infection-induced cytopathic effects after a 3–5-day incubation period at 37 °C and 5% CO_2_ in a humid cell culture incubator.

Samples were tested for anti-SARS-CoV-2 spike-specific antibodies with the Anti-SARS-CoV-2-ELISA IgG or Anti-SARS-CoV-2 QuantiVac-ELISA IgG test system from Euroimmun (Luebeck, Germany) run on the Euroimmun Analyzer I-2P according to the manufacturer's instructions, as described previously [27, 32].

### Statistical analysis

Data were analyzed by using IBM SPSS Statistics 27. In the comparative analysis between COVID-19 subjects and the control group, patient characteristics were analyzed by the t-test when variables were normally distributed. For categorical variables, Chi-squared and Fisher’s exact tests were used. Sperm parameters were analyzed using the Mann–Whitney U test, since sperm parameter values are not normally distributed. Sperm series analysis was completed using the Friedman test. Effect size was calculated using Cochrane’s Q test for categorical variables and Kendell’s W. Regarding the effect size, absolute values ranging from 0.01–0.09, 0.10–0.29, 0.30–0.49, and ≥ 0.50 represent negligible, small, moderate, and strong effects, respectively. All statistical analyses were two-sided, and *p*-values ≤ 0.05 were considered statistically significant.

## Results

The first ejaculate examination was performed 4.4 days (on average) after the onset of symptoms. The mean time interval between the first and second and the first and third ejaculate examinations was 13.4 and 76.6 days, respectively. The mean time interval between the onset of symptoms and the second and third samples was 17.9 and 81.7 days, respectively.

All subjects only had a mild case of COVID-19 and reported the following symptoms: headache (*n* = 20), cough (*n* = 16), fatigue (*n* = 15), fever (*n* = 13), loss of taste (*n* = 12), loss of smell (*n* = 11), sore throat (*n* = 10), diarrhea (*n* = 3), sleep disturbances (*n* = 1), joint pain (*n* = 1), testicular pain (*n* = 1), nausea (*n* = 1), and sinusitis (*n* = 1). To treat the symptoms, subjects took either non-steroidal anti-inflammatory drugs (NSAIDs) or paracetamol. None of the subjects required hospitalization. Apart from fatigue and the loss of smell and taste, the subjects’ symptoms lasted an average of 10.3 (1 − 43 range) days. A total of 10 subjects had already been vaccinated against COVID-19 with an EU-approved vaccine at the time of infection, 6 subjects had been vaccinated twice and 4 subjects had received the vaccine booster.

### Case–control comparison

Except for age, which was significantly lower in the control group than in the case group (*p* = 0.039), there were no significant differences in baseline characteristics (Table 1).

**Table 1:** Basic characteristics of subjects, sperm parameters, and virological semen analyses

	**COVID-19** (*n* = 25)	**Control group** (*n* = 12)	***p*-value**
Age (years)	34.9 (26.7–43.1)	26.7 (24.0–34.8)	0.039^a^
Body mass index (kg/m^2^)	24.1 (23.1–26.9)	23.9 (22.3–26.1)	0.977^a^
Smokers	4 (16.0%)	5 (41.7%)	0.088^c^
Diabetes mellitus type 2	0	0	
Hypertension in need of medical treatment	1	0	
Paternity	6 (24.0%)	2 (16.7%)	0.612^c^
Result of fertility treatment	1 (4.0%)	1 (8.3%)	> 0.999^d^
Testicular cancer	1 (4.0%)	1 (8.3%)	> 0.999^d^
Semen volume (ml)	2.8 (2.0–3.9)	3.9 (2.2–5.0)	0.131^b^
Sperm concentration (10^6^/ml)	44.0 (23.0–82.0)	93.5 (23.5–100.0)	0.140^b^
Total sperm number (10^6^/ejaculate)	136.8 (54.0–228.1)	204.9 (83.1–550.0)	0.153^b^
Progressive motility (%)	25.0 (13.5–35.0)	20.0 (10.0–41.3)	0.708^b^
Total motility (%)	40.0 (18.5–40.0)	25.0 (15.0–47.5)	0.658^b^
Normozoospermia (n)	10 (40%)	5 (41.7%)	0.923^c^
Time of abstinence (days)	4 (3.0–6.5)	3.5 (2.3–4.0)	0.204^b^
SARS CoV-2-positive PCR of ejaculates (n)	0	0	
SARS-CoV-2-positive cell cultures of ejaculates (n)	0	0	

Continuous data are presented as the median (Q1–Q3; Q1 = 25th percentile and Q3 = 75th percentile) and categorical data as frequencies (%)

^a^t-test

^b^Mann-Whitney U test

^c^Chi-squared test

^d^Fisher’s exact test

SARS-CoV-2 IgG was detected in the serum of 23/25 subjects in the COVID-19 group after infection. In one subject, the result for SARS-CoV-2 IgG after infection was 0.33 arbitrary units (AU) per ml, which was considered negative. Another subject had a borderline result with a value of 1.03 AU/ml. Among the 12 subjects in the control group, 11 had a negative SARS-CoV-2 IgG results from serum. One subject from the control group had a borderline result (0.88 AU/ml). The sperm parameter values in direct comparison between the case and control groups also showed no significant differences (Table 1). Only 40% of the COVID-19 group and 41.7% of the control group had normozoospermia.

### Serial ejaculate analysis

Serial examination of sperm parameter values in subjects with acute COVID-19 infection showed no statistically significant differences (Table 2).

**Table 2:** Serial sperm parameters and virological analyses in subjects suffering from COVID-19

	**First sample**	**Second sample**	**Third sample**	***p*-value**	**Effect size**
n	23	23	23		
Semen volume (ml)	2.8 (2.5–3.9)	2.6 (1.8–2.9)	3.0 (1.9–3.6)	0.617^a^	0.021^c^
Sperm concentration (10^6^/ml)	46.0 (25.0–86.0)	32.0 (12.0–78.0)	48.0 (26.0–68.0)	0.435^a^	0.036^c^
Total sperm number (10^6^/ejaculate)	150.0 (79.2–232.5)	97.2 (28.0–176.4)	101.4 (45.6–166.6)	0.062^a^	0.121^c^
Progressive motility (%)	25.0 (15.0–35.0)	20.0 (15.0–40.0)	30.0 (15.0–45.0)	0.220^a^	0.066^c^
Total motility (%)	40.0 (20.0–40.0)	30.0 (20.0–50.0)	40.0 (20.0–55.0)	0.144^a^	0.084^c^
Normozoospermia (n)	9 (39.1%)	9 (39.1%)	9 (39.1%)	> 0.999^a^	0.00^b^
Time of abstinence (days)	4 (3.0–6.0)	3 (3.0–4.0)	4 (2.0–5.0)	0.054^a^	0.127^c^
SARS CoV-2-positive PCR of ejaculates (n)	0	0	0		
SARS-CoV-2-positive cell cultures of ejaculates (n)	0	0	0		

Continuous data are presented as the median (Q1–Q3; Q1 = 25^th^ percentile and Q3 = 75^th^ percentile) and categorical data as frequencies (%)

^a^Friedman test

^b^Cochrane’s Q test

^c^Kendall’s W

Subjects with acute COVID-19 infection who were not vaccinated against SARS-CoV-2 also showed no statistically significant differences in the serial examination of sperm parameters (Table 3).

**Table 3:** Serial analysis of sperm parameter values in unvaccinated subjects suffering from COVID-19

	**First sample**	**Second sample**	**Third sample**	***p*-value**
n	12	12	12	
Semen volume (ml)	2.8 (2.3–3.6)	2.3 (1.9–2.8)	2.5 (1.8–3.1)	0.544^a^
Sperm concentration (10^6^/ml)	45.0 (29.3–78.5)	24.0 (5.4–91.5)	42.5 (14.8–73.3)	0.455^a^
Total sperm number (10^6^/ejaculate)	139.6 (57.8–221.7)	69.6 (9.5–175.5)	76.8 (37.6–207.0)	0.174^a^
Progressive motility (%)	25.0 (15.0–33.8)	15.0 (7.5–33.8)	25.0 (15.0–37.5)	0.247^a^
Total motility (%)	30.0 (20.0–40.0)	20.0 (11.8–42.5)	32.5 (20.0–47.5)	0.250^a^
Normozoospermia (n)	2 (16.7%)	2 (16.7%)	3 (25%)	0.819^a^

Continuous data are presented as the median (Q1-Q3; Q1 = 25^th^ percentile and Q3 = 75^th^ percentile) and categorical data as frequencies (%)

^a^Friedman test

There were no significant differences between sperm parameter values in subjects with or without fever (Table 4).

**Table 4:** Serial analysis of sperm parameter values in subjects suffering from COVID-19 with fever

	**First sample**	**Second sample**	**Third sample**	***p*-value**
n	12	12	12	
Semen volume (ml)	2.8 (2.6–3.8)	2.8 (2.3–2.9)	2.6 (1.4–3.5)	0.640^a^
Sperm concentration (10^6^/ml)	50.0 (27.8–84.0)	29.5 (12.0–75.0)	50.0. (26.3–73.3)	0.262^a^
Total sperm number (10^6^/ejaculate)	146.8 (71.8–221.7)	73.4 (29.4–195.3)	97.5 (55.0–155.8)	0.097^a^
Progressive motility (%)	30.0 (17.5–40.0)	17.5 (8.3–37.5)	30.0. (17.5–45.0)	0.135^a^
Total motility (%)	40.0 (22.5–78.8)	25.0 (11.8–47.5)	40.0. (22.5–58.8)	0.081^a^
Normozoospermia (n)	5 (41.7%)	5 (41.7%)	5 (41.7%)	> 0.999^a^

Continuous data are presented as median (Q1–Q3; Q1 = 25^th^ percentile and Q3 = 75^th^ percentile) and categorical data as frequencies (%)

^a^Friedman test

### SARS-CoV-2 RNA and infectivity analysis

SARS-CoV-2 RNA was not detected by qRT-PCR in any of the semen samples analyzed in this study (Table 1). None of the semen samples obtained in the acute phase of infection showed the presence of infectious particles as determined by cell culture inoculation.

## Discussion

COVID-19 is a disease caused by SARS-CoV-2 that has been spreading since 2019 and is characterized by respiratory symptoms and suspected of having a detrimental effect on male fertility [33]. Although the disease can still be fatal, mild disease courses predominate in people of reproductive age [1]. Therefore, the present study investigated the impact of mild COVID-19 on male fertility.

SARS-CoV-2 RNA was not detected in the ejaculates of our population during the acute disease period nor after recovery, which is in agreement with the majority of research published on this topic [8–12, 14, 15, 21]; however, a few research groups have been able to demonstrate the detection of SARS-CoV-2 in ejaculate [16, 17, 25]. A recently published meta-analysis has shown that neither the severity of the disease nor the age of the patient have an influence on the presence of SARS-CoV-2 in the ejaculate, while the length of the time interval between the onset of the disease and testing seems to have an effect [26]. However, the authors speculated that the detection of SARS-CoV-2 in the ejaculate may also have resulted from contamination [26]. In the present study, the average time from the onset of symptoms to ejaculation was 4.4 days, which is very low compared with the other studies. Temiz et al. hypothesized that drugs "tested out" for COVID-19 therapy may also be causative for the detection of SARS-CoV-2 in the ejaculate, as they may have impaired the blood-testis barrier [12]; however, most studies did not provide detailed information on the therapy used [13, 17]. Subjects from the present study did not take any medication besides NSAIDs and paracetamol. Although only a few studies were able to detect SARS-CoV-2 in ejaculate by PCR [16, 17, 25], none involved cell culture inoculation to detect infectious particles.

The presence of infectious particles is a prerequisite for pathogen transmission as Feldmann [34] has pointed out. Thus, the question of whether SARS-CoV-2 should be considered a risk in terms of sexual transmission cannot be answered without examination for infectious particles. In our cohort, neither SARS-CoV-2 RNA nor infectious particles were found in the samples from the acute phase of infection. Therefore, it would be of great benefit to follow up cell culture inoculation experiments with samples from patients with mild and severe COVID-19.

In the present study, we demonstrated that sperm parameter values of the acutely affected COVID-19 subjects with a mild disease course did not differ from those of a control group, even when only the unvaccinated subjects were analyzed. In contrast, Enikeev et al. showed that sperm motility and sperm morphology were significantly impaired in acutely infected COVID-19 patients compared with a control group [18]; however, this study involved only hospitalized subjects who had a moderate or severe course of disease, whereas our subjects had only mild symptoms. In our article published in May 2020, we already suspected that the impact of COVID-19 on male fertility is dependent on the severity of the disease [8]. This suspicion was confirmed by the research of Gacci et al., in which hospitalized COVID-19 subjects had significantly lower total sperm counts than non-hospitalized COVID-19 subjects [17]. In contrast, Ruan et al. found that sperm quality of COVID-19 patients was significantly lower than that of an age-matched control group, regardless of disease severity, even 80 days after disease onset [14]. The fact that the results on the influence of COVID-19 on sperm quality initially appear contradictory may also be due to the fact that the pathogenesis has not yet been conclusively clarified. The idea that damage to the seminiferous tubules and spermatogenesis is directly caused by the virus is considered very unlikely for the vast majority of cases [35]. Rather, it is likely that inflammation-related cytokine/chemokine dysregulation, fever, and drugs could lead to dysfunction of Leydig and Sertoli cells, altering the gonadal hormonal axis and impairing the antioxidant defense system of seminal fluid, ultimately leading to impaired spermatogenesis [35, 36]. Although this is a very interesting aspect, it was not further investigated in the present study.

The present study found no significant differences in the serial examinations of ejaculate parameter values at 4.4, 17.9, and 81.7 days after symptom onset. In contrast, Guo et al. demonstrated significant improvements in ejaculate parameters in a serial examination 75 and 106 days after COVID-19 infection [19]; however, the cohort was composed of subjects with mild, moderate and severe courses of disease but was not analyzed group-wise due to the small number of subjects (*n* = 22) [19]. Fraietta et al. also published a serial analysis of ejaculate parameters in COVID-19-affected individuals with primarily mild disease courses but with shorter time intervals of 7, 14, and 21 days and, matching our data, did not find any significant differences [20].

For couples with a desire to have children, the serial observation is relevant, especially in the context of fertility treatments, as the question is often asked whether a therapy break should be taken after COVID-19. For couples in which the man had a mild course of COVID-19, this question is irrelevant.

Only 40% (versus 41.7% in the control group) of subjects from the COVID-19 group had normozoospermia, which could be due to the lack of exclusion criteria regarding andrological and internal medicine history. As such, one subject in each of the control and case groups in this study had a history of testicular carcinoma. One subject in each group had received infertility treatment due to reduced male fertility, and 16.0% (COVID-19 group) and 41.7% (control group) of the subjects were smokers, respectively.

In this regard, our work differs from other studies such as that of Temiz et al., which excluded any previous andrological disease [12].

A limitation of the study is the lack of hormonal analysis and andrological examination. In addition, the study population is relatively small, which is due to the necessity of ejaculate collection in the acute stage of the disease being rejected by many potential participants.

Strengths of the study include the serial study design and ejaculates being analyzed as early as 4.4 days after the onset of symptoms. In addition, this is the first study that analyzed male fertility after COVID-19 in subjects that had already been vaccinated against COVID-19.

## Conclusion

It can be concluded that SARS-CoV-2 cannot be detected in the ejaculate of males who have a mild case of COVID-19. A mild course of disease does not appear to affect male fertility as determined by semen parameter analysis.

## Data Availability

The datasets generated during the current study are available from the corresponding author upon reasonable request.

## Abbreviations

ACE2: Angiotensin-converting enzyme 2
AU: Arbitrary units
COVID-19: Coronavirus disease 2019
NSAID: Non-steroidal anti-inflammatory drugs
RT-PCR: Reverse transcription-polymerase chain reaction
SARS-CoV-2: Severe acute respiratory syndrome coronavirus type 2
TMPRSS2: Transmembrane protease serine 2
WHO: World Health Organization
